# Subchronic Exposure to Aripiprazole Subtly Impacts on Rodents' Sperm Quality and Fertility

**DOI:** 10.1111/bcpt.70200

**Published:** 2026-02-03

**Authors:** Maria Joana Nogueira de Moura, Renata Gleysiane Sousa de Felix, Aline Gabriele Gomes da Silva, Ana Beatriz Silva Angelo, Caio César Araújo dos Santos, Artemia Kelly Holanda Pereira, Michelly Fernandes de Macedo, Cibele dos Santos dos Borges

**Affiliations:** ^1^ Laboratory of Tissue and Development Biology – Center for Biological and Health Sciences – CCBS Federal Rural University of the Semi‐Arid Region – UFERSA Mossoro Brazil; ^2^ Multicenter Postgraduate Program in Biochemistry and Molecular Biology University of the State of Rio Grande do Norte – UERN Mossoro Brazil; ^3^ Laboratory for Diagnostics in Veterinary Clinical Pathology, Department of Animal Sciences Federal Rural University of the Semi‐Arid Region – UFERSA Mossoro Brazil

## Abstract

Aripiprazole, a third‐generation antidepressant/antipsychotic drug, acts on serotonergic and dopaminergic receptors and is widely prescribed for mental disorders, such as depression and schizophrenia. Studies indicate that this class of drugs can impact directly on sperm quality and fertility. Then, this study evaluated the effects of subchronic exposure to aripiprazole on sperm quality and fertility of adult male Wistar rats. Thirty‐six rats were divided into three groups: control, 3 mg/kg and 6 mg/kg of aripiprazole orally exposed for 28 days. Our results showed that aripiprazole significantly increased seminal vesicle weight at a dose of 6 mg/kg. Progressive sperm motility was reduced, and there was an increase in the diameter of the seminiferous tubules at a dose of 3 mg/kg. Fertility test was reduced at a dose of 6 mg/kg. These findings suggest that although aripiprazole is effective in treating mental disorders, it may have subtle adverse effects on reproduction, especially on sperm quality and fertility, raising concerns about its indiscriminate use at doses higher than those tested.

## Plain Language Summary

Aripiprazole, a third‐generation antidepressant/antipsychotic, is widely prescribed for mental disorders, and studies indicate a possible impact on the male reproductive system. Thus, our data show that aripiprazole significantly impacts sperm motility and fertility, suggesting that although effective in treating mental disorders, it may have subtle adverse effects on reproduction, raising concerns about indiscriminate use at doses higher than those tested.

## 1 Introduction

Major depressive disorder (MDD) is one of the most severe and prevalent psychiatric conditions globally [1]. The World Health Organization recognizes it as a primary gateway to other major organic diseases [2]. Although it is more common in women [3, 4], MDD affects several biological systems in both sexes, including the central nervous system [5, 6], immune system [7], endocrine system [8] and gastrointestinal system [9]. MDD is characterized by a dysfunction in the neurotransmission of 5‐hydroxytryptamine (5‐HT), the serotonin [10]; the transporter protein SERT plays an important role in this pathway, with different serotonergic receptors, such as 5‐HT1A, 5‐HT1B and 5‐HT2A [11].

The main treatments include drugs, such as selective serotonin reuptake inhibitors (SSRIs) and serotonin and norepinephrine reuptake inhibitors (SNRIs) [1], and psychotherapy [12]. These medications work by blocking the reuptake of neurotransmitters such as serotonin and norepinephrine but can cause adverse effects such as nausea, insomnia and sexual dysfunction [13]. Serotonin also influences reproductive function by interacting with sex steroids [14]. Furthermore, combination therapies of reuptake inhibitors associated with antipsychotic drugs have been applied when previous treatment is ineffective [15].

Antipsychotics, in turn, are widely used in the treatment of schizophrenia, a disease whose global prevalence of 0.4% represents a significant problem for families and society, because these patients have high mortality rates and comorbid medical conditions [16].

Aripiprazole, a third‐generation atypical antipsychotic, introduced into the pharmaceutical market in 2002, is used to treat disorders such as schizophrenia, bipolar disorder and MDD, which was the subject of these studies. The oral dosage ranges from 5 mg to 30 mg/day and is available in 2, 5, 10, 15, 20 and 30 mg tablets [17]. This medication acts as a partial agonist at dopamine D2 and serotonin 5‐HT1A receptors and as an antagonist at 5‐HT2A receptors [18, 19, 20], offering a profile of action that minimizes the extrapyramidal side effects associated with first‐generation antipsychotics [21]. Aripiprazole has a long half‐life [22], with effects that could influence the male reproductive system [23], because it is a system that responds directly to neuroendocrine control, which exerts distinct control functions mediated by neurotransmitters and sex hormones [24]. Thus, the present study explores the complex interrelationship between aripiprazole treatment and sperm quality, considering the implications for male fertility.

## 2 Materials and Methods

### 2.1 Animals

Thirty‐six adult male *Wistar* rats were used for drug experimentation and 15 adult females for mating (90 days old) from the Central Animal Facility of the State University of Rio Grande do Norte—UERN and were kept in the Animal Facility of the Department of the School of Health Sciences (UERN). The animals were kept under controlled conditions (21°C ± 2°C), 12‐h light–dark cycle and had free access to food and water. All experimental procedures were conducted in accordance with the Experimental Principles of the Ethics Committee of the State University of Rio Grande do Norte, CEEA/UERN No. 00621. The animals were monitored daily by the team veterinarian for any signs of pain or anxiety. All animals were considered fit until the end of the experiment. The study was conducted in accordance with the *Basic & Clinical Pharmacology & Toxicology* policy for experimental and clinical studies [25].

### 2.2 Experimental Design

**FIGURE 1:**
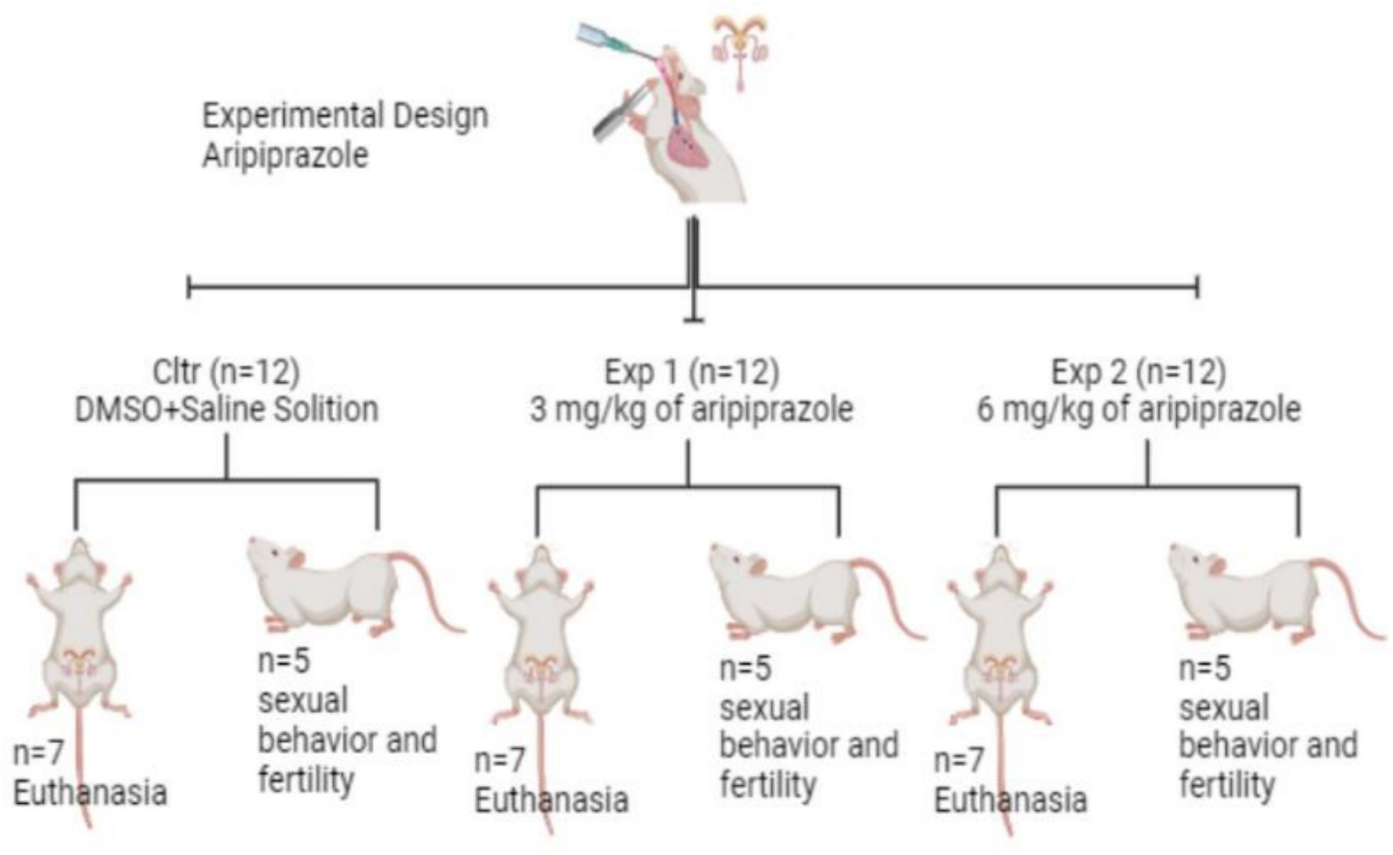
Illustration of the distribution of experimental groups (*n* = 36) divided into control group (Ctrl = DMSO + saline solution): Experimental Group 1 (3.0 mg/kg of aripiprazole) and Experimental Group 2 (6.0 mg/kg of aripiprazole).

The animals (*n* = 36; Figure 1) were randomly distributed among the three experimental groups (simple randomization, *n* = 12/group): control group (Ctrl), 3.0 mg/kg of aripiprazole group and 6.0 mg/kg of aripiprazole group. Experimental groups received 2 mL/kg as a maximum volume of treatment. The control group (Ctrl) received only the vehicle (33% of the volume in dimethylsulfoxide [DMSO] and 67% of the volume in saline solution). The drug was administered orally, and the treatment period was 28 days, a period comprising part of spermatogenesis (spermatocytotoxicity), which is important for sperm formation as well as sperm quality because it includes sperm transit time. The drug doses used in this experimental model were based on the protocol adopted by Sanabria et al., which correspond to the available oral doses of 10 and 30 mg/day of treatment.

After the treatment period, the study was divided into two experimental phases: Phase 1, animals destined for organ collection, sperm quality and histological evaluation (*n* = 7 animals/group), and Phase 2, animals destined for sexual behaviour, as well as fertility assessment (*n* = 5 animals/group). The number of animals per group was chosen based on the minimum number of animals required to perform parametric statistical tests for each parameter evaluated. Furthermore, all animals were used in experiments, with no exclusion or inclusion after the start of treatments. The animals in each experimental group were kept identified in their respective cages (*n* = 3/cage), totalling four cages per experimental group. All identifications were checked daily to avoid any confusion between animals and experimental groups by a single researcher assigned to this task. After the treatment period, the animals were weighed and euthanized. All euthanasia procedures were performed via intraperitoneal injection with a dose of 240 mg/kg of ketamine and 80 mg/kg of xylazine.

### 2.3 Organ Weights

On the 29th day of the experiment, the 21 males (*n* = 7/group) were weighed and euthanized. After euthanasia, blood was collected (9:00 and 11:30 AM) and the right testis, epididymis, seminal vesicle (full and empty, without the coagulating gland) and ventral prostate were dissected and weighed. The left testis and epididymis were fixed for histological analyses. The right testis and epididymis were used for sperm parameters (motility, morphology and count).

### 2.4 Histological Evaluation of the Testis

The left testis was fixed by immersion in Modified Davidson's Solution (MDF—composed of 53% distilled water; 2% formalin 37%; 10% glacial acetic acid 99.7% and 35% ethanol 100%). After 24 h, the testis was dehydrated in increasing concentrations of ethanol, diaphanized in xylene and embedded in paraffin. Then, the tissues were sectioned (3–5 μm), mounted on a glass slide and stained with haematoxylin and eosin (HE) to evaluate testicular morphology and histopathology.

The histopathological evaluation was performed using an optical microscope with a digital camera attached (Leica DMLB). It evaluated 100 sections of the seminiferous tubules of each animal, observing the appearance of the epithelium, lumen content and interstitium of the testicles so that possible morphological lesions of these organs could be classified according to specific guidelines for toxicological studies [26].

The diameter of the seminiferous tubules and the thickness of the germinal epithelium were evaluated using 10 sections of seminiferous tubules per animal (*n* = 7/group) in tubules in Stage IX of spermatogenesis. This was done using a Leica DMLB microscope with a magnification of 200× and analysed with Zeiss AxioVision, Version 4.7.2.

To evaluate the dynamics of the spermatogenic process, the relative frequency of the stages was estimated: I–VI (presents two generations of spermatids), VII–VIII (presents mature spermatids located at the edge of the lumen), IX–XIII (presents only one generation of spermatids) and XIV (presents secondary spermatocytes). One hundred cross sections of seminiferous tubules per animal were evaluated, most regularly and circularly possible. The relative frequency of the stages estimates the rhythm or duration of the spermatogenic process [23].

The number of Sertoli cell nuclei and the volume of Leydig cells were also determined. The Sertoli cell nuclei were determined in histological sections in 10 seminiferous tubules per rat testis in Stage VII of spermatogenesis. The nuclear volume of Leydig cells was measured by randomly selecting 50 circular or elliptical cells; their diameters (D) were measured, and the volume was obtained using the formula: V = (D × π)/6.

### 2.5 Sperm Assessment

Sperm motility was assessed using the cauda epididymis, which was isolated, and sperm obtained, as described by Borges et al. Briefly, under a stereomicroscope, sperm were released from the proximal cauda of the epididymis and transferred into 1 mL of PBS (82 g sodium chloride; 10.5 g sodium phosphate; 3.55 g monobasic sodium phosphate; 1 L of distilled/deionized water) enriched with 1 g of BSA (fetal bovine serum) and transferred to a BOD incubator at 34°C for 3 min. A 10‐μL aliquot of the sperm suspension was immediately transferred to a Neubauer chamber, and using a light microscope (400× magnification), 100 sperm were counted and classified as Type A (mobile with progressive movement), Type B (mobile without progressive movement) and Type C (non‐mobile).

For morphological evaluation, sperm was obtained from the same solution used for the motility procedure. A 100‐μL aliquot of the solution containing spermatozoa was transferred to 900 μL of 10% formalin saline solution, and smears were made on histological slides, left to dry in the open air for 30 min and then observed under a light microscope (400× magnification). It was evaluated 200 spermatozoa per animal, and the morphological abnormalities found were classified into two categories: head abnormalities (without characteristic curvature, isolated or duplicated) or tail abnormality (curled, broken, folded and isolated) [25].

### 2.6 Sperm Count and Sperm Transit Time

Testicular spermatids resistant to homogenization (Stage 19 of spermiogenesis) were counted as follows: testes (*n* = 7/group) were decapsulated and homogenized in a mixture of 5 mL of 0.9% NaCl containing 0.5% Triton X‐100, followed by sonication for 30 s. After a 10‐fold dilution, a sample was transferred to Neubauer chambers (four fields per animal), and mature spermatids at Stage 19 were counted under a light microscope. To calculate sperm production per day (SPD), the number of spermatids at Stage 19 was divided by 6.1, which is the number of days of the cycle during which these spermatids are present in the seminiferous epithelium. Likewise, the head/body and tail of the epididymis were cut into small fragments with scissors and homogenized following the testicular protocol. The transit time of the sperm through the epididymis was determined by dividing the number of sperm in each portion by the PDE. The total sperm count in the epididymal cauda was the sum of the values obtained by sperm count and the values obtained by counting during the procedure to obtain motile sperm [26].

### 2.7 Experiment 2: Sexual Behaviour and Natural Mating

#### 2.7.1 Sexual Behaviour of Males

Fifteen nulliparous adult females (*n* = 5/group) in pro‐oestrus/natural oestrus were used. To perform the behaviour test, the male rats were first isolated for 10 min, and then a female was introduced into the box. The evaluations were performed in the dark cycle of the light/dark cycle. For 40 min, the following parameters were recorded: times of first mount, first intromission, first ejaculation, first mount after ejaculation, post‐ejaculation intromission and the number of intromissions until the first ejaculation, the number of post‐ejaculation intromissions, and the number of ejaculations. After the behavioural assessment, the female remained in the box with the male throughout the night, and the following morning, a vaginal lavage was performed to confirm whether or not insemination had occurred.

#### 2.7.2 Fertility Assessment

On the 20th day after the behavioural test described above, the females (*n* = 15) were weighed and euthanized to assess fertility. The uterus and ovaries were exposed by laparotomy, and the following parameters were collected and assessed: number of corpora lutea, implantations and reabsorptions, weight of the gravid ovary, fetal weights and sexing and placental weight. Therefore, the following parameters were calculated: pregnancy rate or index: (number of females that achieved pregnancy/number of potentially fertile females that were mated) × 100; fertility potential (implantation efficiency): (implantation sites/corpora lutea) × 100; pre‐implantation loss rate: [(number of corpora lutea − number of implantations)/number of corpora lutea] × 100; and post‐implantation loss rate: [(number of implantations − number of live fetuses)/number of implantations] × 100.

### 2.8 Statistical Analysis

Data normality was verified using the Shapiro–Wilk test. For data with normal distribution, one‐way ANOVA and Tukey or Dunnett tests were applied to compare means. Non‐parametric results were analysed using the Mann–Whitney or Kruskal–Wallis tests, with comparisons of means made using Dunn's test. Results were expressed as mean ± standard error of the mean (SEM), and differences were considered significant at *p* ≤ 0.05. Statistical analyses were performed using GraphPad InStat software (Version 5).

## 3 Results

The effects of aripiprazole treatment on reproductive organ weights showed an increase in absolute and relative weight of the seminal vesicle in the 6.0 mg group (0.96 g ± 0.02 g and 333.5 ± 9.81 mg/100 g, respectively) compared to the control group (0.77 ± 0.03 g and 279.30 ± 7.51 mg/100 g). However, the body weight and vital target organs did not show significant differences (*p* > 0.05; Table 1).

**TABLE 1:** Absolute and relative weight of the reproductive organs of animals exposed to 3.0 and 6.0 mg/kg of aripiprazole.

Parameters	Control (*n* = 7)	3.0 mg/kg (*n* = 7)	6.0 mg/kg (n = 7)
Final body weight (g)	277.00 ± 7.86	271.20 ± 4.90	288.40 ± 5.44
Organ weight			
Testicle (g)	1.61 ± 0.03	1.66 ± 0.04	1.72 ± 0.02
Epididymis (g)	0.61 ± 0.01	0.63 ± 0.02	0.66 ± 0.01
Prostate (g)	0.29 ± 0.03	0.29 ± 0.02	0.31 ± 0.03
Full seminal vesicle (g)	0.77 ± 0.03^a^	0.84 ± 0.07^ab^	0.96 ± 0.02^b^
Empty seminal vesicle (g)	0.31 ± 0.01	0.33 ± 0.03	0.32 ± 0.01
Pituitary (mg)	8.04 ± 0.42	6.74 ± 0.42	6.13 ± 0.93
Thyroid (g)	0.04 ± 0.01	0.04 ± 0.01	0.02 ± 0.00
Relative weight of organs			
Testicle (mg/100 g)	584.70 ± 18.18	613.80 ± 10.95	597.70 ± 13.47
Epididymis (mg/100 g)	222.70 ± 9.53	233.60 ± 5.51	228.60 ± 5.45
Prostate (mg/100 g)	105.50 ± 12.24	109.10 ± 6.02	106.90 ± 10.06
Full seminal vesicle (mg/100 g)	279.30 ± 7.51^a^	310.80 ± 21.80^ab^	333.50 ± 9.8^b^
Empty seminal vesicle (mg/100 g)	112.60 ± 6.04	121.10 ± 0.33	112.20 ± 4.83
Pituitary (mg/100 g)	2.91 ± 0.14	2.48 ± 0.12	2.14 ± 0.34
Thyroid (mg/100 g)	12.72 ± 3.26	16.42 ± 4.27	9.91 ± 1.90

*Note:* Data expressed as mean ± SEM. Different letters express statistical difference (*p* < 0.05). ANOVA followed by Tukey test.

**FIGURE 2:**
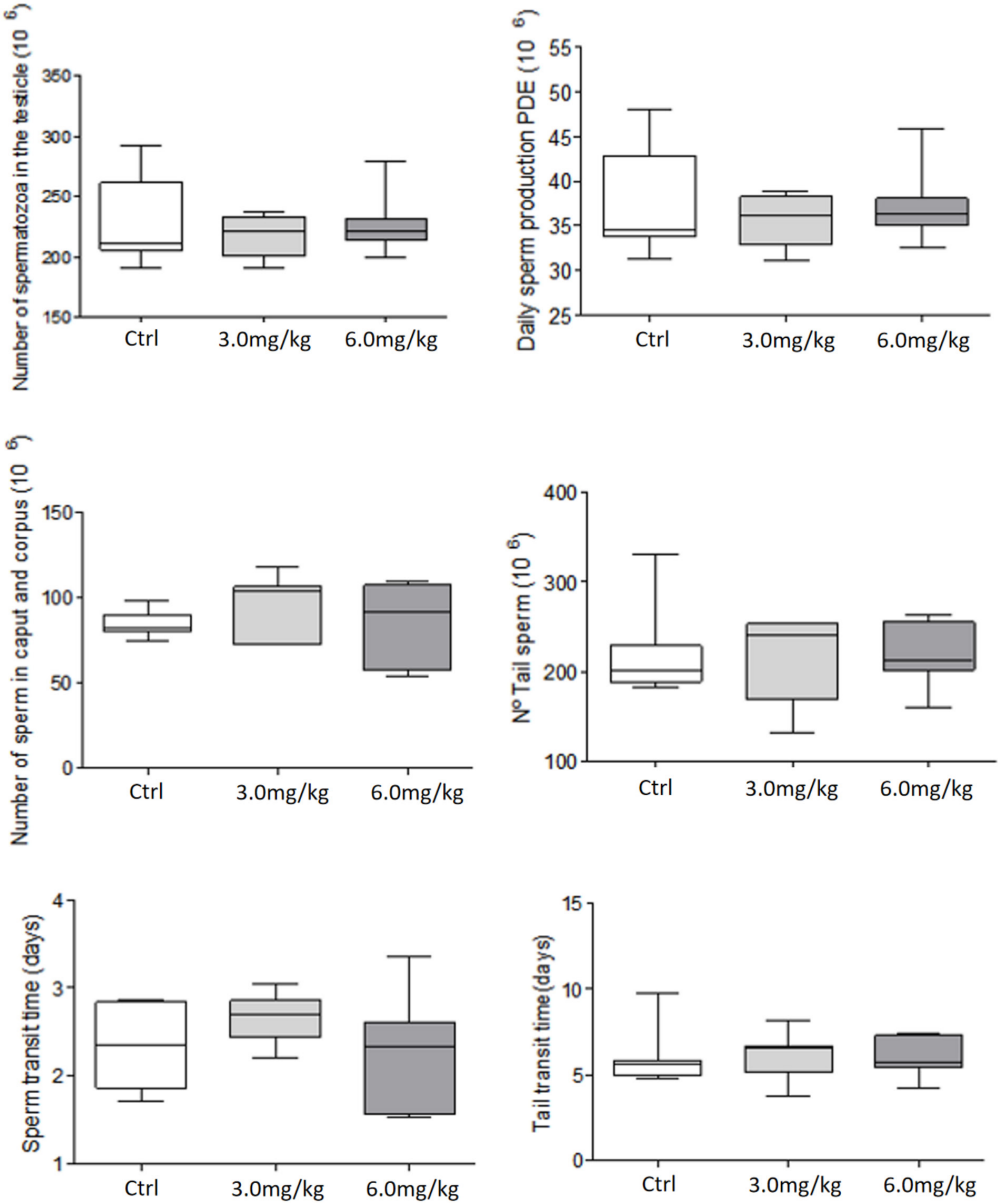
Analysis of daily sperm production (DSP), number of sperm in the testis, head–body and tail of the epididymis, as well as epididymal transit time in Wistar rats treated with different concentrations of aripiprazole. Values expressed as mean + SEM (parametric ANOVA followed by Tukey test), *p* > 0.05.

**FIGURE 3:**
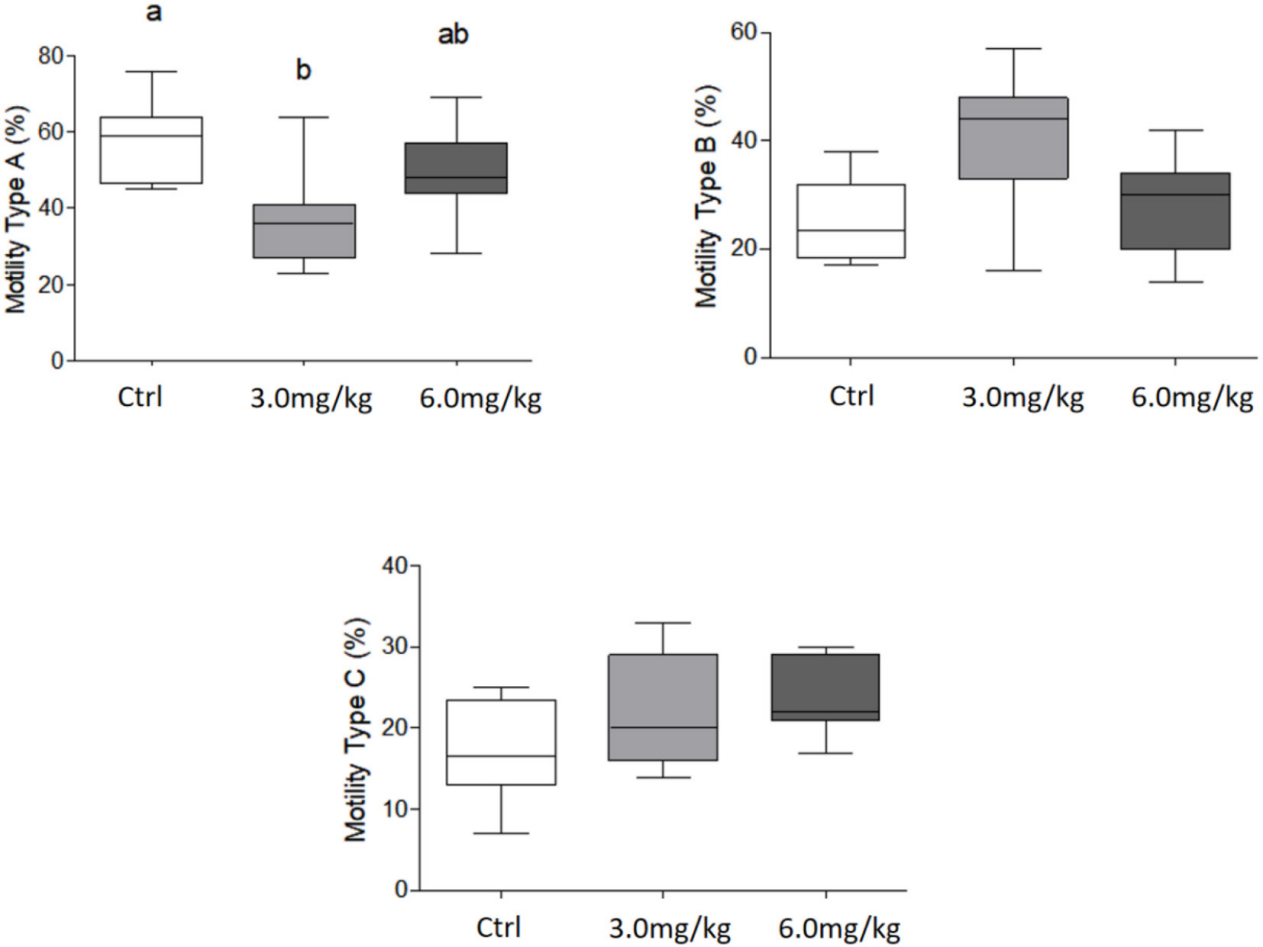
Analysis of sperm motility in the epididymal fluid of Wistar rats treated with different concentrations of aripiprazole. Classification of spermatozoa into spermatozoa with progressive movement—Type A. Motile spermatozoa without progressive movement—Type B. Immobile spermatozoa—Type C. Values expressed as mean + SEM (Kruskal–Wallis test followed by Dunn's test). Different letters express statistical difference (*p* < 0.05).

Regarding sperm parameters, no changes were observed on daily sperm production in the testis. In the epididymis, although the sperm transit time, sperm concentration and morphology were not altered (*p* > 0.05; Figure 2), a significant reduction in sperm motility was observed in the 3.0 mg group, with a lower percentage of motile with progressive movement sperm when compared to the control group (57.7% × 37.7%; Figure 3).

**FIGURE 4:**
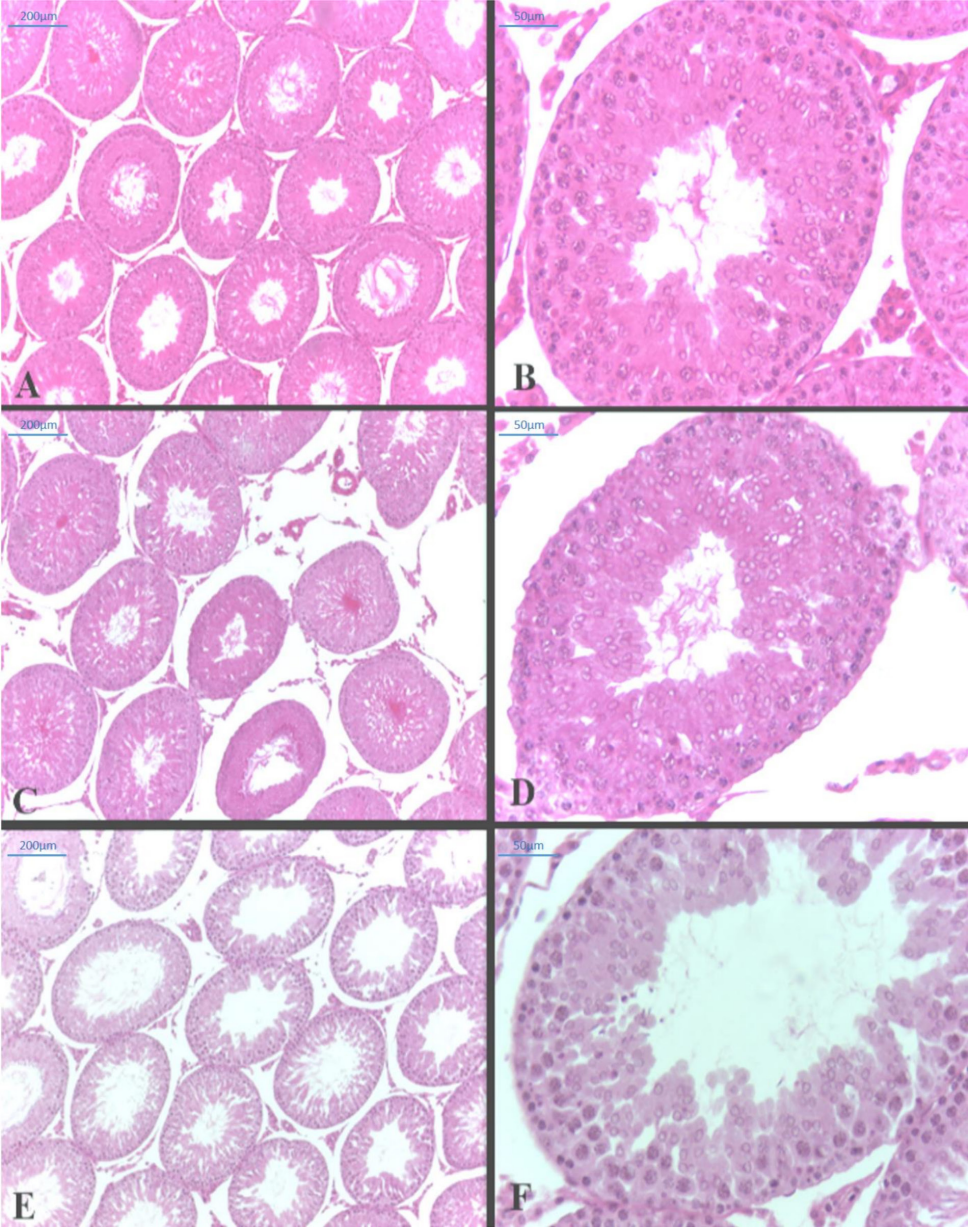
Photomicrograph of cross sections of adult rat testes, HE staining. (A,B) Control Group. (C,D) 3 mg/kg group and (E,F) 6 mg/kg group. Magnitude: 10× and 40× magnification.

The histopathological assay (Table 2) revealed a significant increase in the diameter of the seminiferous tubules in the 3.0 mg group (321.10 ± 5.76 μm) compared to control group (285.10 ± 5.93 μm). Figure 4 showed no alterations on epithelial height and Leydig cells volume, as well as in the Sertoli cell number.

**TABLE 2:** Histopathological and morphometric analysis of the testis, as well as spermatogenic dynamics of Wistar rats treated with different concentrations of aripiprazole.

Parameters	Control (*n* = 7)	3.0 mg/kg (*n* = 7)	6.0 mg/kg (*n* = 7)
Testicular histopathology			
Normal tubules (%)	86.86 ± 2.67	85.00 ± 3.38	87.57 ± 2.49
Depleted tubules (%)	5.00 ± 1.41	4.85 ± 1.73	3.14 ± 0.63
Tubules with vacuoles (%)	5.00 ± 1.71	4.57 ± 1.21	3.67 ± 1.25
Tubules with eosinophilia (%)	1.00 ± 0.00	2.00 ± 0.00	1.25 ± 0.25
Tubules with degeneration (%)	5.25 ± 2.59	5.75 ± 4.11	6.00 ± 3.18
Tubules with other abnormalities (%)	2.14 ± 0.55	2.80 ± 0.58	2.33 ± 0.61
Spermatogenic dynamics			
I–VI (%)	32.14 ± 2.49	32.71 ± 2.58	31.57 ± 2.59
VII–VIII (%)	31.14 ± 3.13	31.14 ± 1.88	31.57 ± 2.51
IX–XIII (%)	28.29 ± 2.03	30.43 ± 1.97	29.57 ± 3.15
XIV (%)	8.429 ± 0.72	5.71 ± 0.83	7.29 ± 1.11
Morphometric assays			
Tubule diameter (μm)	285.10 ± 5.93^a^	321.10 ± 5.76^b^	294.50 + 6.09^ab^
Epithelium cell height (μm)	85.97 ± 3.38	93.12 ± 4.57	89.57 ± 2.47
Leydig cell volume (μm)	107.90 ± 6.24	106.60 ± 13.71	103.20 ± 3.29
Number of Sertoli cells	30.29 ± 2.55	29.67 ± 2.29	24.77 ± 2.13

*Note:* Data expressed as mean + SEM. Different letters express statistical difference (*p* < 0.05). ANOVA followed by Tukey test.

Sexual behaviour (Table 3) was affected, with a significant decrease in the number of mounts (16.0 + 3.2 × 6.0 + 1.5) and an increase in the time of first mount (120.6 s + 34.4 s × 47.00 + 20.54 s) and time of first intromission (171.8 s + 46.42 s × 66.60 + 38.210 s) in animals in the 3.0 mg group compared to the control group.

**TABLE 3:** Analysis of sexual behaviour of Wistar rats treated with different concentrations of aripiprazole.

Parameters	Control (*n* = 7)	3.0 mg/kg (*n* = 7)	6.0 mg/kg (*n* = 7)
Latency for first mount(s)	47.00 + 20.54^a^	120.60 + 34.46^b^	71.80 + 18.13^ab^
Latency to first intrusion(s)	66.60 + 38.210^a^	171.80 + 46.42^b^	115.8 + 33.30^ab^
Latency until first ejaculation(s)	881.00 + 125.90	1160.00 + 98.00	1225.00 + 135.10
First post‐ejaculation latency(s)	1202.00 + 208.0	1320.00 + 142.5	1437.00 + 88.43
Latency of first post‐ejaculation intromission(s)	1202.00 + 208.0	1320.00 + 142.5	1457.00 + 96.70
Number of mounts	16.00 + 3.24^a^	6.00 + 1.45^b^	7.00 + 2.59^ab^
Number of intrusions	21.40 + 2.25	19.60 + 3.17	16.20 + 2.94

*Note:* Data expressed as mean + SEM. Different letters express statistical difference (*p* < 0.05). ANOVA followed by Tukey test.

After the gestational period, the laparotomy and fertility assay were performed. Our results showed a significant reduction in fertility potential of the 6.0 mg group (83.00% + 6.95%) when compared to the control group (93.00 ± 2.39%). The other reproductive parameters, such as female weight, gravid uterus weight, fetus and placenta weight, number of implantations, number of resorptions and post‐implantation losses, did not show significant differences between the groups (*p* > 0.05) (Table 4).

**TABLE 4:** Fertility test of Wistar rats treated with different Aripiprazole concentrations after natural mating with females no treated.

Parameters	Control (*n* = 7)	3.0 mg/kg (*n* = 7)	6.0 mg/kg (*n* = 7)
Body weight of females (g)^a^	304.5 ± 11.88	287.90 ± 8.00	286.00 ± 14.60
Weight of the uterus with fetuses (g)^a^	60.74 ± 6.18	57.84 ± 5.50	68.90 ± 7.70
Number of corpora lutea^a^	13.00 ± 0.84	14.20 ± 1.24	17.17 ± 2.24
Number of deployments^a^	12.60 ± 0.75	12.00 ± 1.00	13.80 ± 0.66
Number of resorptions^a^	2.33 ± 0.67	1.25 ± 0.25	3.00 ± 1.50
Fetal weights (g)^a^	3.65 ± 0.12	3.39 ± 0.49	3.43 ± 0.29
Placental weight (g)^a^	0.51 ± 0.01	0.59 ± 0.06	0.48 ± 0.01
Fertility potential (%)^b^	93.00 ± 2.39^a^	85.40 ± 6.32^ab^	83.00 ± 6.95^b^
Pre‐implantation loss (%)^b^	7.00 ± 2.39	14.60 ± 6.32	17.00 ± 6.95
Post‐implantation loss (%)^b^	11.40 ± 5.73	10.20 ± 3.61	10.83 ± 6.80

^a^ Data expressed as mean + SEM ANOVA, followed by Tukey test.

^b^ Values expressed as percentage (chi‐square test). Different letters express statistical difference (*p* < 0.05).

## 4 Discussion

MDD has become a concern for the World Health Organization (WHO), as its incidence is increasing, both in men and women. There is a range of medications available for pharmacological treatment. However, in this study, we chose aripiprazole, a third‐generation antipsychotic that promises to reduce extrapyramidal effects and, consequently, reduce side effects. In our study, we present altered results of aripiprazole exposure on sperm motility, testicular morphology, sexual behaviour and fertility in adult male rats in different doses of this drug.

The reproductive organ weights are considered markers of reproductive toxicity [27]. The results of our study showed that aripiprazole, when administered during 28 days at 6.0 mg/kg, caused an increase in the seminal vesicle with secretion and no changes in the seminal vesicle without secretion, which may indicate a possible change in the neuroendocrine control of this gland, because these accessory glands are much more susceptible to hormonal or neurotransmitter fluctuations [28, 29].

Considering that the seminal vesicle is highly innervated by the autonomic nervous system [30], it is possible that aripiprazole influenced the contractile functioning of cells in this organ and, consequently, decreased the elimination of vesicular fluid [31] once aripiprazole acts as a partial agonist at dopamine D2 and serotonin 5‐HT1A receptors [18, 19, 20]. Hsieh et al. [32] previously showed that activation of peripheral 5‐HT1A receptors can inhibit seminal vesicle contraction. Furthermore, Nojimoto et al. [33] showed that high doses of serotonin and norepinephrine reuptake inhibitors can have an impact by decreasing the contractile activity of the seminal vesicle, promoting greater fluid accumulation. Moreover, finally, it is known that contraction of the seminal vesicle mediated by dopaminergic receptor stimuli acts indirectly, promoting noradrenaline release from sympathetic nerve endings [34], which reinforces our hypothesis of a reduction in fluid release only at the highest dose.

Regarding testicular parameters, there was no significant difference in any treatment when compared to the control group. However, it is interesting to highlight in this study the daily sperm production in groups 3.0 and 6.0 mg and also the number of sperm in the caput/corpus of the epididymis of the same groups. These parameters are important indicators of male fertility, because the number of mature spermatids in the epididymis is directly related to the daily sperm production in the testis [35, 36]. Although not significant, these results together may reflect the action of aripiprazole on serotonergic and dopaminergic receptors, as observed by the weight of the seminal vesicle [37], especially at the highest dose.

In the present study, no significant changes were observed in the sperm transit time in the epididymis of animals evaluated in the treatments of 3.0 and 6.0 mg groups. Sperm transit time is regulated by androgen hormones and contractile activity of the smooth muscle present around the duct [38, 39, 40]. Kempinas et al. showed that the delay in epididymal transit time does not alter the sperm fertility capacity, but when this time is accelerated, fertility is compromised. This loss occurs because the time available for the processes required to acquire fertile capacity is reduced [41, 42]. Knowing that the epididymis is highly responsive to noradrenergic stimulation [43], poorly mediated by aripiprazole [44], it is possible that the effects of the treatment did not have such an impact on this organ, especially in the caput/corpus. However, the responsiveness of the epididymis is region specific, in which the epididymal cauda region is the most responsive to neurotransmitters. This further reinforces our hypothesis, because the epididymal cauda, although not statistically, was different from the caput/corpus region, with a slight reduction in sperm concentration. Indeed, this statement can be reinforced by the findings of sperm motility, once the 3.0 mg treatment showed a significant difference in Type A motility (mobile sperm with progressive movement), which was reduced in relation to the control. It is important to know that sperm motility is one of the most commonly used parameters to assess sperm fertile capacity [45, 46].

The evaluation of fertility parameters showed that males exposed to the 6.0 mg group presented a significant decrease in fertility potential compared to the control and the 3.0 mg group and showed an increase in the pre‐implantation loss rate. These results may have occurred due to the impact on the sperm maturation process in the experimental groups [42, 47].

It is known that the evaluation of sperm morphology provides valuable information on the dose–response of a chemical substance [48] and also is related to the spermatogenesis process [49] as well as the histopathological evaluation, the most sensitive parameter for detecting the effects on male reproductive function [42]. Our data showed no alterations in both parameters, reinforcing the testicular data (weight and daily sperm production). On the other hand, it was observed that there was an increase in the diameter of the seminiferous tubule from the 3.0 mg group when compared to the control group. Because there was no change in the number of Sertoli cells, a parameter that generally determines the diameter of the seminiferous tubules [50], this change can be correlated to a slight increase in the daily production of sperm.

Another important parameter to evaluate when using drugs that can impact neuronal control is sexual behaviour [37]. Because aripiprazole acts directly on dopaminergic and serotonergic receptors [51], the animals in the Exp 1 group presented changes in sexual behaviour, as the number of mounts was statistically reduced. Another point observed, which expresses a biological result, was the evaluation of the time of the first mount and intromission, in which the animals in both experimental groups with aripiprazole were increased. The testosterone levels are essential for the performance of sexual behaviour [52]. Although not measured in the present study, our data on Leydig cell volume indicate that there was no change in hormone production, once the Leydig cells are the primary cells involved in steroidogenesis in the testis and that any interference with their activity can trigger changes in the production and/or action of steroid hormones, especially testosterone [53].

Thus, our results suggest that aripiprazole, although effective for the treatment of depressive and psychoactive diseases, has deleterious effects, albeit subtle, on reproductive parameters in this experimental model, suggesting an impact on sperm quality and, consequently, on fertility, which raises concerns about the indiscriminate use of such substances in doses higher than those used in the present study, and, taking into account the high reproductive efficiency of rodents, the impacts on humans may be even greater than those presented.

## 5 Conclusion

This study indicates that both treatments with aripiprazole, at doses of 3.0 and 6.0 mg/kg, caused damage to the male reproductive system, albeit in a subtle manner. It was evident that aripiprazole impacts sexual behaviour, the spermatogenesis process and fertility in animals.

## Funding

This work was supported by the Federal Rural University of Semi‐Arid Region (23091.014593/2019‐02 and 23091.005800/2023‐42), the Coordination for the Improvement of Higher Education Personnel – Brazil (CAPES) – Finance Code 001 and the National Council for Scientific and Technological Development.

## Conflicts of Interest

The authors declare no conflicts of interest.

## Data Availability

The data that support the findings of this study are available on request from the corresponding author.
